# The Enzymatic Activity of the nsp14 Exoribonuclease Is Critical for Replication of MERS-CoV and SARS-CoV-2

**DOI:** 10.1128/JVI.01246-20

**Published:** 2020-11-09

**Authors:** Natacha S. Ogando, Jessika C. Zevenhoven-Dobbe, Yvonne van der Meer, Peter J. Bredenbeek, Clara C. Posthuma, Eric J. Snijder

**Affiliations:** a Molecular Virology Laboratory, Department of Medical Microbiology, Leiden University Medical Center, Leiden, The Netherlands; Loyola University Chicago

**Keywords:** replicase, nonstructural protein, RNA synthesis, proofreading, reverse genetics, guanine-N7-methyltransferase, nidovirus

## Abstract

Coronaviruses (CoVs) stand out for their large RNA genome and complex RNA-synthesizing machinery comprising 16 nonstructural proteins (nsps). The bifunctional nsp14 contains 3′-to-5′ exoribonuclease (ExoN) and guanine-N7-methyltransferase (N7-MTase) domains. While the latter presumably supports mRNA capping, ExoN is thought to mediate proofreading during genome replication. In line with such a role, ExoN knockout mutants of mouse hepatitis virus (MHV) and severe acute respiratory syndrome coronavirus (SARS-CoV) were previously reported to have crippled but viable hypermutation phenotypes. Remarkably, using reverse genetics, a large set of corresponding ExoN knockout mutations has now been found to be lethal for another betacoronavirus, Middle East respiratory syndrome coronavirus (MERS-CoV). For 13 mutants, viral progeny could not be recovered, unless—as happened occasionally—reversion had first occurred. Only a single mutant was viable, likely because its E191D substitution is highly conservative. Remarkably, a SARS-CoV-2 ExoN knockout mutant was found to be unable to replicate, resembling observations previously made for alpha- and gammacoronaviruses, but starkly contrasting with the documented phenotype of ExoN knockout mutants of the closely related SARS-CoV. Subsequently, we established *in vitro* assays with purified recombinant MERS-CoV nsp14 to monitor its ExoN and N7-MTase activities. All ExoN knockout mutations that proved lethal in reverse genetics were found to severely decrease ExoN activity while not affecting N7-MTase activity. Our study strongly suggests that CoV nsp14 ExoN has an additional function, which apparently is critical for primary viral RNA synthesis and thus differs from the proofreading function that, based on previous MHV and SARS-CoV studies, was proposed to boost longer-term replication fidelity.

**IMPORTANCE** The bifunctional nsp14 subunit of the coronavirus replicase contains 3′-to-5′ exoribonuclease (ExoN) and guanine-N7-methyltransferase domains. For the betacoronaviruses MHV and SARS-CoV, ExoN was reported to promote the fidelity of genome replication, presumably by mediating a form of proofreading. For these viruses, ExoN knockout mutants are viable while displaying an increased mutation frequency. Strikingly, we have now established that the equivalent ExoN knockout mutants of two other betacoronaviruses, MERS-CoV and SARS-CoV-2, are nonviable, suggesting an additional and critical ExoN function in their replication. This is remarkable in light of the very limited genetic distance between SARS-CoV and SARS-CoV-2, which is highlighted, for example, by 95% amino acid sequence identity in their nsp14 sequences. For (recombinant) MERS-CoV nsp14, both its enzymatic activities were evaluated using newly developed *in vitro* assays that can be used to characterize these key replicative enzymes in more detail and explore their potential as target for antiviral drug development.

## Abstract (precis)

The bifunctional nsp14 subunit of the coronavirus replicase contains 3′-to-5′ exoribonuclease (ExoN) and guanine-N7-methyltransferase domains. For the betacoronaviruses MHV and SARS-CoV, ExoN was reported to promote the fidelity of genome replication, presumably by mediating a form of proofreading. For these viruses, ExoN knockout mutants are viable while displaying an increased mutation frequency. Strikingly, we have now established that the equivalent ExoN knockout mutants of two other betacoronaviruses, MERS-CoV and SARS-CoV-2, are nonviable, suggesting an additional and critical ExoN function in their replication. This is remarkable in light of the very limited genetic distance between SARS-CoV and SARS-CoV-2, which is highlighted, for example, by 95% amino acid sequence identity in their nsp14 sequences. For (recombinant) MERS-CoV nsp14, both its enzymatic activities were evaluated using newly developed *in vitro* assays that can be used to characterize these key replicative enzymes in more detail and explore their potential as target for antiviral drug development.

## INTRODUCTION

RNA viruses commonly exhibit high mutation rates, a feature attributed to the relatively poor fidelity of their RNA-dependent RNA polymerase (RdRp) and the fact that nucleotide incorporation errors go uncorrected. This lack of proofreading contributes to the generation of “quasispecies” populations, clouds of genome sequence variants that are subject to continuous natural selection (1–3). On the one hand, their genetic heterogeneity allows RNA viruses to rapidly adapt to changing circumstances, in order to overcome environmental challenges such as host switching, antiviral drug treatment, or host immune responses (4, 5). On the other hand, the accumulation of an excessive number of deleterious mutations can result in “error catastrophe” and, consequently, in the extinction of a viral species (6–8). In order to balance these opposing principles, RNA viruses are thought to operate close to their so-called error threshold, while balancing the interdependent parameters of replication fidelity, genome size, and genome complexity (9, 10). This interplay is thought to have restricted the expansion of RNA virus genome sizes, which are below 15 kb for most RNA virus families (10–12).

**FIG 1:**
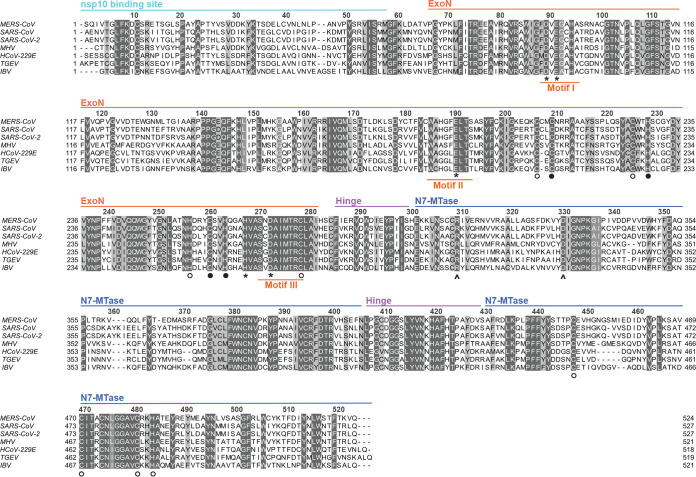
Alignment of nsp14 amino acid sequences from selected coronaviruses. Sequences of the ExoN and N7-MTase domains in MERS-CoV (NC-019843), SARS-CoV (NC_004718), SARS-CoV-2 (NC_045512.2), MHV (NP_045298), HCoV-229E (NC_002645), TGEV (AJ271965), and IBV (NP_040829) were used for the analysis. The domains indicated at the top are based on the SARS-CoV-nsp14 secondary structure (PDB 5NFY) (21). Fully conserved residues are shown with white letters on dark gray (above 70% conservation), whereas partially conserved residues are displayed with lighter shades of gray. Catalytic residues and residues involved in formation of zinc fingers are marked with asterisks and circles, respectively. Filled circles indicate zinc fingers targeted by mutagenesis (Fig. 2A), while arrowheads identify the two N7-MTase domain residues mutated to generate the MTase negative control used in biochemical assays. The alignment was generated using Clustal Omega (104) and edited using Jalview version 2.11 (105).

The largest RNA virus genomes currently known are found in the order *Nidovirales*, which includes the family *Coronaviridae* and also the recently discovered planarian secretory cell nidovirus (PSCNV) (12, 13), which has the largest RNA genome identified thus far (41.1 kb). One of the molecular mechanisms potentially driving the unprecedented expansion of nidovirus genomes was discovered about 17 years ago, during the in-depth bioinformatics analysis of the genome and proteome of the severe acute respiratory syndrome coronavirus (SARS-CoV). During this analysis, Alexander Gorbalenya and colleagues identified a putative 3′-to-5′ exoribonuclease (ExoN) signature sequence in the N-terminal domain of nonstructural protein 14 (nsp14), a subunit of the large replicase polyprotein encoded by CoVs and related large-genome nidoviruses. Strikingly, this ExoN domain was found to be lacking in the replicases of nidoviruses with small(er) genomes (specifically, arteriviruses), and therefore, it was proposed that the enzyme may provide a form of proofreading activity that could have promoted the expansion of large nidoviral genomes to their current size (10–12, 14). Comparative sequence analysis with cellular homologs classified the nidoviral/CoV ExoN domain as a member of the superfamily of DEDDh exonucleases, which also includes the proofreading domains of many DNA polymerases as well as other eukaryotic and prokaryotic exonucleases (15). These enzymes catalyze the excision of nucleoside monophosphates from nucleic acids in the 3′-to-5′ direction, using a mechanism that depends on two divalent metal ions and a reactive water molecule (16–18). Five conserved active-site residues arranged in three canonical motifs (I, II, and III) (Fig. 1) orchestrate ExoN activity (14, 19–21). Additionally, the domain incorporates two zinc finger (ZF) motifs (10), ZF1 and ZF2 (Fig. 1), that were hypothesized to contribute to the structural stability and catalytic activity, respectively, of ExoN (20).

The predicted 3′-to-5′ exoribonuclease activity of the CoV ExoN domain was first confirmed *in vitro*, in biochemical assays using recombinant SARS-CoV nsp14 and different synthetic RNA substrates (19). Originally, residues D90/E92 (motif I), D243 (motif II), and D273 (motif III) were identified as putative active-site residues of SARS-CoV ExoN (14, 19). However, the SARS-CoV nsp14 crystal structure revealed E191 rather than D243 to be the acidic active residue in motif II, demonstrating that ExoN is in fact a DEEDh enzyme (20). By using reverse genetics for the alphacoronavirus human coronavirus 229E (HCoV-229E), Minskaia et al. demonstrated that inactivation of the ExoN active site results in failure to recover infectious viral progeny (19). Interestingly, a quite different phenotype was described for the corresponding ExoN knockout mutants of two betacoronaviruses, mouse hepatitis virus (MHV) and SARS-CoV. While ExoN inactivation decreased replication fidelity in these viruses, conferring a 'mutator phenotype', the mutants were viable, both in cell culture (22, 23) and in animal models (24). These findings suggested that ExoN may indeed be part of an error correction mechanism. Subsequently, the ability of ExoN to excise 3′-terminal mismatched nucleotides from a double-stranded RNA (dsRNA) substrate was demonstrated *in vitro* using recombinant SARS-CoV nsp14 (25). Furthermore, this activity was shown to be strongly enhanced (up to 35-fold) by the addition of nsp10, a small upstream subunit of the CoV replicase (26). The two subunits were proposed to operate, together with the nsp12 RdRp, in repairing misincorporations that may occur during CoV RNA synthesis (21, 27). In cell culture, MHV and SARS-CoV mutants lacking ExoN activity exhibit increased sensitivity to mutagenic agents like 5-fluorouracil (5-FU), compounds to which the wild-type virus is relatively resistant (28, 29). Recently, ExoN activity was also implicated in CoV RNA recombination, as an MHV ExoN knockout mutant exhibited altered recombination patterns, possibly reflecting its involvement in other activities than error correction during CoV replication and subgenomic mRNA synthesis (30). Outside the order *Nidovirales*, arenaviruses are the only other RNA viruses known to employ an ExoN domain, which is part of the arenavirus nucleoprotein and has been implicated in fidelity control (31) and/or immune evasion, the latter by possibly degrading viral dsRNA (32, 33). Based on results obtained with transmissible gastroenteritis virus (TGEV) and MHV ExoN knockout mutants, the CoV ExoN activity was also suggested to counteract innate responses (34, 35).

In the meantime, CoV nsp14 had been proven to be a bifunctional protein by the discovery of a guanine-N7-methyltransferase (N7-MTase) activity in its C-terminal domain (36) (Fig. 1). This enzymatic activity was further corroborated *in vitro*, using biochemical assays with purified recombinant SARS-CoV nsp14. The enzyme was found to be capable of methylating cap analogues or GTP substrates, in the presence of *S*-adenosylmethionine (SAM) as methyl donor (36, 37). The N7-MTase was postulated to be a key factor for equipping CoV mRNAs with a functional 5′-terminal cap structure, as guanine-N7-methylation is essential for cap recognition by the cellular translation machinery (25). Although the characterization of the nsp14 N7-MTase active site and reaction mechanism was not completed, alanine scanning mutagenesis and *in vitro* assays with nsp14 highlighted several key residues (Fig. 1) (36, 38, 39). Moreover, crystal structures of SARS-CoV nsp14 in complex with its nsp10 cofactor (PDB entries 5C8U and 5NFY) revealed several unique structural and functional features (20, 21). These combined structural and biochemical studies confirmed that the two enzymatic domains of nsp14 are functionally distinct (36) and physically independent (20, 21). Still, the two activities are structurally intertwined, as it seems that the N7-MTase activity depends on the integrity of the N-terminal ExoN domain, whereas the flexibility of the protein is modulated by a hinge region connecting the two domains (21).

Coronaviruses are abundantly present in mammalian reservoir species, including bats, and pose a continuous zoonotic threat (40–43). To date, seven CoVs that can infect humans have been identified, and among these, severe acute respiratory syndrome coronavirus 2 (SARS-CoV-2) is currently causing an unprecedented pandemic outbreak. The previous zoonotic CoV to emerge, in 2012, was the Middle East respiratory syndrome coronavirus (MERS-CoV) (44). Due to its transmission from dromedary camels and subsequent nosocomial transmission, MERS-CoV continues to circulate and cause serious human disease, primarily in the Arabian Peninsula (45). Occasional spread to other countries has also occurred, including an outbreak with 186 confirmed cases in South Korea in 2015 (46–48). Like SARS-CoV, SARS-CoV-2, and MHV, MERS-CoV is classified as a member of the genus *Betacoronavirus*, although it belongs to a different lineage (subgenus) of that cluster (49, 50). The current lack of approved therapeutics and vaccines to prevent or treat CoV infections, as well as the general threat posed by emerging CoVs, necessitates the further in-depth characterization of CoV replication and replicative enzymes.

In this context, the quite different phenotypes described for ExoN knockout mutants of other CoVs (see above) prompted us to study the importance of this enzyme for MERS-CoV replication. To this end, using both reverse genetics and biochemical assays with recombinant nsp14, we engaged in an extensive site-directed mutagenesis study, targeting all active-site motifs of the MERS-CoV ExoN domain. Strikingly, in contrast to what was observed for two other betacoronaviruses, MHV and SARS-CoV, our studies revealed that ExoN inactivation severely impacts MERS-CoV replication, resulting in failure to recover viable progeny. While completing our MERS-CoV nsp14 studies, given the developing pandemic, we also evaluated the impact of ExoN inactivation (using a D90A/E92A ExoN motif I double mutant) on SARS-CoV-2 replication and viability. Given the close phylogenetic relationship between SARS-CoV and SARS-CoV-2, reflected for example in 95% nsp14 amino acid identity (51), we were highly surprised to find that—as for our MERS-CoV ExoN knockout mutants—it was not possible to rescue viable progeny for this SARS-CoV-2 mutant in which two key residues of the ExoN active site were mutated. Our biochemical evaluation of MERS-CoV nsp14 mutants suggested that this phenotype is not caused by inadvertent side effects of ExoN inactivation on N7-MTase activity. Our combined data suggest that CoV ExoN and/or nsp14 plays a more direct and fundamental role in CoV RNA synthesis than merely safeguarding the long-term fidelity of replication and can thus be considered a prominent target for the development of antiviral drugs.

## RESULTS

### ExoN inactivation is lethal for MERS-CoV.

**FIG 2:**
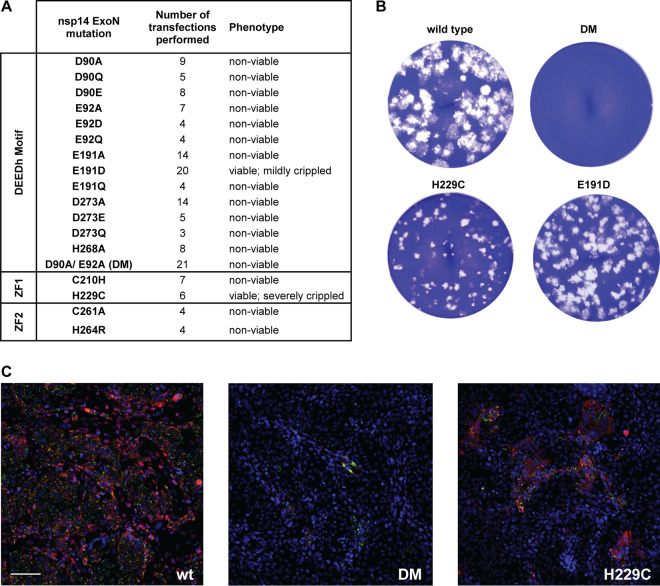
MERS-CoV ExoN knockout mutants are nonviable. (A) Phenotype of MERS-CoV nsp14 ExoN mutants used in this study, scored at 2 dpt. (B) Comparison of plaque phenotype of selected ExoN mutants in HuH7 cells. Plaque assays were performed using supernatants harvested from transfected cell cultures at 3 dpt, which were diluted 10^−4^ for wt and mutant E191D and used in undiluted form for the D90A/E92A ExoN knockout double mutant (DM) and the H229C ZF1 mutant. (C) Immunolabeling (2 dpt) of cell cultures consisting of a mixture of (nonsusceptible) BHK-21 cells transfected with *in vitro*-made full-length MERS-CoV RNA and susceptible (DPP4-expressing) Vero cells used to amplify any infectious progeny released from the transfected BHK-21 cells. (Left) wt virus; (middle) DM mutant; (right) H229C mutant. Cells were labeled for dsRNA (green) and nsp4 (red). Bar, 100 μm.

Previous studies into CoV ExoN function involved its biochemical characterization (based almost exclusively on the SARS-CoV version of the enzyme) and the phenotypic analysis of (predicted) ExoN knockout virus mutants, generated using reverse genetics approaches. The latter studies yielded replication-incompetent ExoN knockout mutants for the alphacoronaviruses HCoV-229E (19) and TGEV (34). However, the equivalent mutants of the betacoronaviruses SARS-CoV and MHV-A59 were somewhat crippled but clearly viable, while displaying a 15- to 20-fold-increased mutation rate (22, 23). An alignment of CoV nsp14 amino acid sequences is presented in Fig. 1, including that of SARS-CoV-2, which emerged in humans during the course of this project. It highlights the key motifs/residues of the two enzymatic domains of nsp14, as well as other structural elements, like the nsp10 binding site, the hinge region connecting the ExoN and N7-MTase domains, and three previously identified nsp14 zinc finger domains (20, 21). The alignment also illustrates the generally high degree of nsp14 sequence conservation across different CoV (sub)genera. In the present study, we targeted all five predicted active-site residues of the MERS-CoV ExoN domain (D90, E92, E191, D273, and H268) by replacing them with alanine as well as more conservative substitutions (D to E or Q; E to D or Q). This yielded a total of 14 ExoN active-site mutants (Fig. 2A), including the D90A/E92A motif I double mutant (DM), which was frequently used as a prototypic viable ExoN knockout mutant in MHV and SARS-CoV studies.

A bacterial artificial chromosome (BAC)-based MERS-CoV reverse genetics system (52, 53), based on the sequence of the EMC/2012 isolate of MERS-CoV (54), served as the starting point to evaluate our ExoN mutants by transfection of full-length RNA that was obtained by *in vitro* transcription using T7 RNA polymerase. Transcripts were electroporated into BHK-21 cells, which lack the DPP4 receptor required for natural MERS-CoV infection (55, 56) but are commonly used to launch engineered CoV mutants because of their excellent survival of the electroporation procedure (19, 22, 23, 34, 52, 57). As BHK-21 cells have a severely compromised innate immune response (58), they also seemed an appropriate cell line to launch ExoN knockout mutants in case the enzyme was needed to counter innate immunity (34, 35). To amplify any progeny virus released, transfected BHK-21 cells were mixed with either innately immune-deficient (Vero) or -competent (HuH7) cells, which are both naturally susceptible to MERS-CoV infection.

In stark contrast to what was previously described for MHV and SARS-CoV, mutagenesis of ExoN active-site residues was found to render MERS-CoV nonviable. When cell cultures (BHK-21 mixed with Vero or HuH7 cells) were analyzed by immunofluorescence microscopy at 2 days posttransfection (dpt), using antibodies recognizing dsRNA and nsp4, abundant signal and virus spread were always observed for wild-type MERS-CoV and the E191D mutant. For the other 13 mutants tested, some labeling was generally observed in a low percentage (less than 2%) of the cells (Fig. 2C, middle), usually in the form of single positive cells or a few positive cells together. However, virus spreading across the dish was not observed regardless of whether Vero or HUH7 cells were used for propagation of recombinant virus progeny, unless reversion had first occurred (see Discussion). In line with these observations, infectious progeny could not be detected when supernatants of transfected-cell cultures harvested at 3 or 6 dpt were analyzed in plaque assays (Fig. 2A and B and data not shown). The single exception was the mutant carrying the conservative E191D replacement in ExoN motif II (Fig. 1), which was alive but somewhat crippled (discussed in more detail below). These results were consistent across a large number of independent repeats (>10 for several of the mutants) (Fig. 2A), performed with RNA transcribed from independently engineered (and fully sequenced) duplicate full-length cDNA clones. The nonviable phenotype of MERS-CoV ExoN mutants in both cell types suggests that innate immune responses did not influence the outcome of these experiments.

Coexpression of the viral nucleocapsid (N) protein has been reported to boost the transfection efficiency of full-length CoV RNA transcripts (59–61). In an ultimate attempt to rescue progeny for our nonviable MERS-CoV mutants, an *in vitro*-made mRNA expressing the MERS-CoV N protein gene mutant was cotransfected with nsp14 mutant or wild-type (wt) full-length RNA. This modification indeed somewhat increased the BHK-21 transfection rate, as monitored by performing infectious center assays with recombinant wt MERS-CoV-transfected cells (data not shown). However, it did not result in the recovery of infectious progeny for any of six nonviable mutants tested (D90E, E191A, D273A, H268A, DM, and C210H mutants), unless (as was occasionally observed) reversion had first occurred, as confirmed by reverse transcription-PCR (RT-PCR) amplification and sequencing of the nsp14-coding region.

### ExoN inactivation is also lethal for SARS-CoV-2.

**FIG 3:**
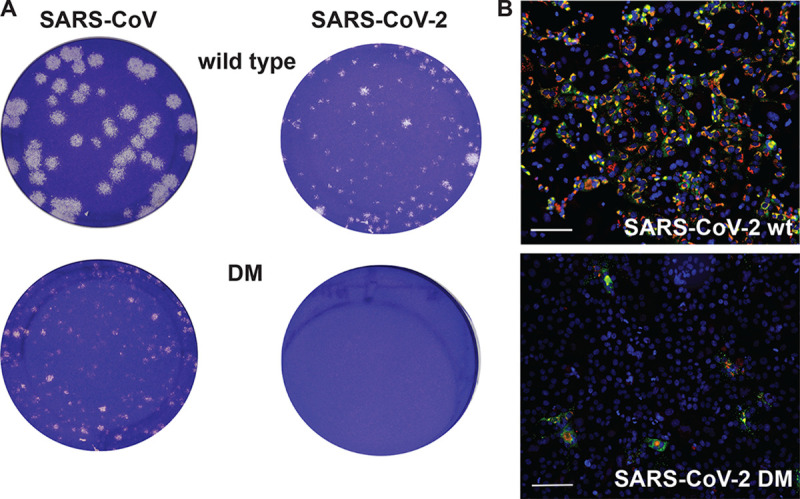
A SARS-CoV-2 ExoN knockout mutant is nonviable. (A) Plaque phenotypes of wt SARS-CoV (left) and SARS-CoV-2 (right) and their corresponding ExoN motif I knockout double mutants (DM; D90A/E92A) in Vero E6 cells. Plaque assays were performed using supernatants harvested from transfected cell cultures at 2 dpt for SARS-CoV and 3 dpt for SARS-CoV-2. Samples were diluted 10^−6^ for SARS-CoV wt, 10^−5^ for SARS-CoV-2 wt and SARS-CoV DM, and 10^−1^ for the SARS-CoV-2 DM mutant. (B) Immunolabeling (2 dpt) as described for Fig. 2C but with Vero E6 cells for amplification of SARS-CoV-2 progeny released from BHK-21 cells transfected with wt (top) or DM (bottom) full-length RNA. Bar, 100 μm.

During the final stage of this study, given the ongoing pandemic, we evaluated whether ExoN inactivation also affects SARS-CoV-2 replication. This was not expected given the viable phenotype of SARS-CoV ExoN knockout mutants and the close relationship between that virus and SARS-CoV-2 (43, 50), which is for example reflected in nsp14 amino acid sequences being ∼95% identical between the two viruses (51). Four independently engineered and fully sequenced SARS-CoV-2 BAC clones were used to transcribe full-length RNA carrying the D90A/E92A double mutation in ExoN motif I, which has been used in many studies with MHV and SARS-CoV ExoN knockout mutants (23, 28, 29). These transcripts were transfected into SARS-CoV N protein-expressing BHK-21 cells (59), in the presence or absence of a synthetic mRNA expressing the SARS-CoV-2 N protein. Subsequently, the transfected cells were mixed (1:1) with Vero E6 cells to support propagation of any viable progeny virus. Wild-type SARS-CoV and its corresponding (viable) ExoN knockout mutant (D90A/E92A; DM) were used as positive controls in these experiments. Surprisingly, using culture supernatant harvested at 3 or 6 dpt, viable progeny of the SARS-CoV-2 DM mutant could not be recovered in any of the four independent repeats (Fig. 3). In contrast, for SARS-CoV, at 2 dpt, both the wild-type and ExoN knockout mutant were already producing abundant progeny, although the plaque phenotype and titers (1.5- to 2-log difference) of the ExoN knockout mutant were clearly reduced compared to those of the parental virus (Fig. 3A). As for the corresponding MERS-CoV mutant, immunofluorescence microscopy revealed some signal for the SARS-CoV-2 DM mutant in a low percentage of the cells by 2 dpt (Fig. 3B), but virus spreading was not observed up to 6 dpt. These results highlight that the impact of ExoN inactivation on general viability can be very different in different viruses, even between two closely related CoVs.

### ExoN inactivation abrogates detectable MERS-CoV RNA synthesis.

**FIG 4:**
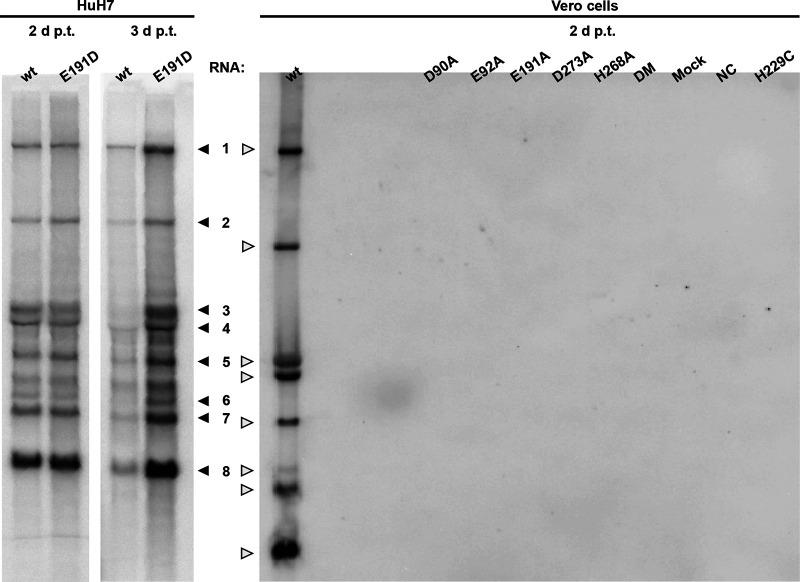
Impact of ExoN inactivation on intracellular MERS-CoV RNA synthesis. In-gel hybridization analysis of intracellular RNA isolated after 2 or 3 dpt of transfected BHK-21 cells, which were subsequently mixed with HuH7 or Vero cells, as indicated. Purified RNA was separated in an agarose gel and probed with a radiolabeled oligonucleotide probe recognizing the MERS-CoV genome and subgenomic mRNAs.

For a selection of MERS-CoV ExoN knockout mutants, intracellular RNA was isolated from transfected cell cultures at 2 dpt and analyzed by hybridization and reverse transcription-quantitative PCR (RT-qPCR) to more rigorously measure viral RNA synthesis (Fig. 4). In this analysis, a nonviable MERS-CoV mutant with an in-frame 100-amino-acid deletion in the nsp12 RdRp domain was used as a negative control (NC) for viral RNA synthesis, in order to assess and correct for the detection of any residual full-length RNA transcript that might still be present at this time point after transfection. Upon direct in-gel hybridization analysis using a ^32^P-labeled probe recognizing the 3′ end of all viral mRNAs, the characteristic nested set of MERS-CoV transcripts could be detected only for the E191D mutant and the wt control (Fig. 4). Even after a 28-day exposure of the phosphor imager screen (data not shown), signal could not be detected for any of the other mutants.

The lack of detectable MERS-CoV-specific RNA synthesis was further confirmed using RT-PCR assays specifically detecting genomic RNA and subgenomic mRNA3. RNA accumulation was evaluated at 1 and 2 dpt for seven selected ExoN active-site mutants (D90A, D90E, E191A, E191D, D273A, and H268A mutants and DM) using samples from two independent experiments both comprising duplicate transfections for each mutant. Again, MERS-CoV-specific genomic and subgenomic RNA synthesis was detected only for the E191D mutant and the wt virus control (data not shown). For all other mutants, the RT-PCR assays yielded cycle threshold (*C_T_*) values in the range obtained for samples from mock-infected cells and the replication-deficient NC mutant. In conclusion, with the exception of E191D (see below), all our engineered ExoN active-site mutations abrogated detectable viral RNA synthesis, suggesting that in the case of MERS-CoV—and likely also SARS-CoV-2—the enzyme is indispensable for basic productive replication in cell culture.

### Characterization of rMERS-CoV-nsp14-E191D replication kinetics and 5-FU sensitivity.

Among our MERS-CoV ExoN active-site mutants, only the E191D mutant yielded viable progeny (Fig. 2). This mutant appeared to be genetically stable, as the substitution was preserved upon multiple consecutive passages in HuH7 or Vero cells (data not shown). Interestingly, the E191D mutation transforms the DEEDh catalytic motif into the DEDDh motif, which is characteristic of members of the exonuclease family to which the CoV ExoN belongs (62). In fact, when ExoN sequences from different nidovirus taxa were compared (14, 19), the equivalent of E191 was found to alternate between E and D (63), in line with the observation that this mutation is tolerated in MERS-CoV ExoN.

**FIG 5:**
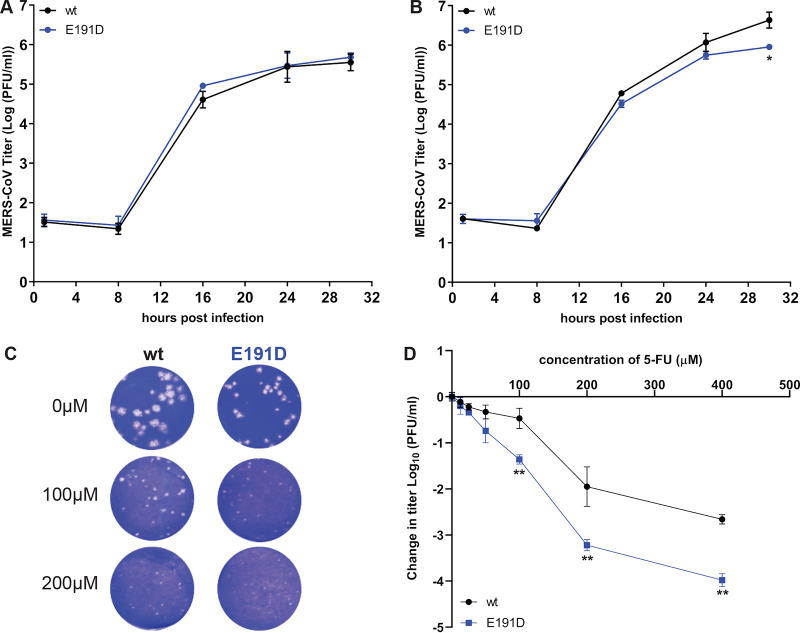
Characterization of growth kinetics of rMERS-nsp14-E191D and its sensitivity to 5-FU treatment. Vero cells (A) or HuH7 cells (B) were infected at an MOI of 3, supernatant was harvested at the indicated time points, and viral progeny titers were measured by plaque assay from two independent experiments using duplicates (*n* = 4; values are means ± standard deviations [SD]). (C) Plaque phenotype in HuH7 cells of rMERS-CoV nsp14-E191D and wt control in the absence or presence of the mutagenic agent 5-FU. (D) Dose-response curve of wt and E191D mutant MERS-CoV in the presence of 5-FU concentrations up to 400 μM (MOI, 0.1; *n* = 4; means ± SD). Statistical significance of the difference with wt virus at each time point (A and B) or concentration (D) was assessed by paired *t* test. *, *P* < 0.05; **, *P* < 0.005.

To characterize the E191D mutant in more detail, its replication and fitness in cell culture were analyzed. Full-length genome sequencing of passage 2 of the E191D mutant virus revealed that it had acquired two additional mutations compared with the recombinant wt control: a synonymous mutation in the nsp2-coding region (U to C at nucleotide [nt] position 2315) and a nonsynonymous mutation (C to A at position 6541), corresponding to an A1235D substitution in the betacoronavirus-specific marker (βSM) domain of nsp3, which has been predicted to be a nonenzymatic domain (64) and is absent in alpha- and deltacoronaviruses (65, 66). Thus, we assumed that any changes in viral replication were likely caused by the E191D mutation in nsp14 ExoN. The same virus stock was used to assess growth kinetics in HuH7 cells (Fig. 5B) and Vero cells (Fig. 5A), which were found to be quite similar for wt and mutant virus. Still, the E191D mutant was found to be somewhat crippled, yielding smaller plaques and somewhat lower progeny titers in HuH7 cells (Fig. 5B and C and 2B) but not in Vero cells (Fig. 5A).

We next examined the sensitivity of E191D and wt virus to the mutagenic agent 5-FU, which is converted intracellularly into a nucleoside analogue that is incorporated into viral RNA (67, 68). Previously, MHV and SARS-CoV ExoN knockout mutants were found to exhibit increased sensitivity to 5-FU treatment, in particular in multicycle experiments, which was attributed to a higher mutation frequency in the absence of ExoN-driven error correction (28). We employed the same assay to assess the phenotype of the E191D mutant in more detail, by performing plaque assays in HuH7 cells in the presence of increasing 5-FU concentrations (Fig. 5C) and by growing mutant and wt virus in the presence of increasing 5-FU concentrations (Fig. 5D). No cytotoxicity was observed in HuH7 cells following treatment with up to 400 μM 5-FU (data not shown).

Plaque assays in HuH7 cells were performed for 3 days, using a standard inoculum of 30 PFU and an increasing amount of 5-FU in the overlay. Similar dose-dependent reductions of plaque size were observed for E191D and wt virus, with E191D virus plaques being barely visible upon treatment with 200 μM 5-FU (Fig. 5C). In an alternative experiment, Huh7 cells were infected at a multiplicity of infection (MOI) of 0.1 and treated with a different 5-FU dose for 30 h, after which progeny virus titers were determined by regular plaque assay. Again, both viruses exhibited similar concentration-dependent decreases of replication (Fig. 5D), although the E191D mutant appeared to be somewhat more sensitive to the mutagenic agent, yielding ∼1-log-lower progeny titers than wt virus upon treatment with 5-FU concentrations between 100 and 400 μM.

Taken together, these experiments demonstrated that overall replication of the E191D mutant is only mildly affected and that it is somewhat more sensitive to 5-FU treatment than wt MERS-CoV. This phenotype is consistent with the bioinformatics-based prediction that a mutant nsp14 carrying this substitution may retain ExoN activity, as is demonstrated below.

### ExoN zinc finger motifs are important for viral replication.

Studies addressing the structural biology and biochemistry of SARS-CoV nsp14 suggested that the two ZF motifs within the ExoN domain contribute to either its structural stability (ZF1) or its catalytic activity (ZF2) (20, 21). Moreover, mutagenesis studies of the MHV and TGEV ZF1 domains supported their importance for viral replication in cell culture (34, 57). To study the impact of similar mutations on MERS-CoV replication, the nsp14 ZF1 and ZF2 domains were targeted with two mutations each, and their impact on virus viability was evaluated as described above. Two ZF1 residues (C210 and H229) were mutated from H to C or vice versa, which could theoretically preserve the zinc-coordinating properties (69, 70). Two residues of the nonclassical ZF2 motif were also substituted (C261A and H264R) to evaluate the ZF2 mutations previously analyzed by Ma et al. (20), leading to disruption of ExoN activity *in vitro*.

The four ZF virus mutants were launched as described above, after which a low level of replication could be observed only for the H229C ZF1 mutant (Fig. 2C), for which the 2-dpt harvest yielded very small plaques (Fig. 2B) and 2- to 4-log-reduced progeny titers, depending on the experiment and time point of harvesting (data not shown). For this mutant, RNA synthesis could not be detected by hybridization analysis (Fig. 4), but synthesis of genomic and subgenomic RNA (mRNA3) was detected by RT-PCR in intracellular RNA samples harvested at 2 dpt (data not shown). A 6-dpt harvest was used for full genome sequencing by next-generation sequencing (NGS), which confirmed the presence of the engineered nsp14 mutation in addition to the appearance of some minor genetic variants (point mutations representing less than 15% of the total population) in different regions of the genome, including the ORF1a domains encoding nsp3, nsp6, nsp8, and nsp9. Taken together, our observations indicate that the H229C mutant is viable but severely crippled. In combination with the fact that the other ZF mutations (C210H in ZF1 and C261A and H264R in ZF2) abolished MERS-CoV replication, our study establishes the importance of both ExoN ZF motifs for MERS-CoV viability.

### Development of a MERS-CoV ExoN activity assay using recombinant nsp14.

**FIG 6:**
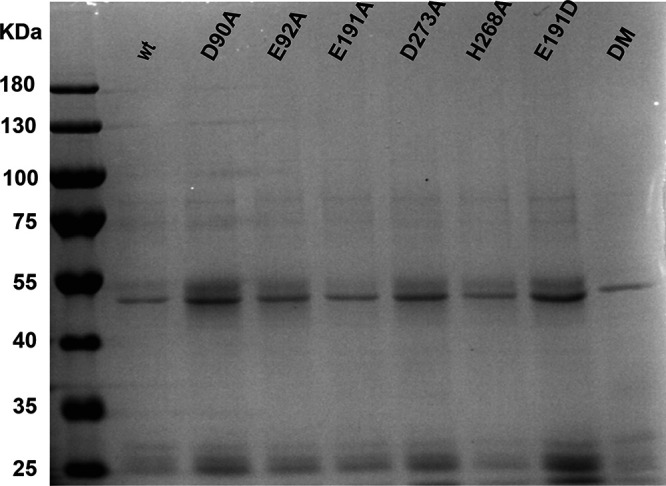
Expression and purification of recombinant MERS-CoV nsp14. N-terminally His-tagged wt and mutant MERS-CoV nsp14 (∼55 kDa) was expressed in E. coli, affinity purified, and analyzed in a 10% SDS-PAGE gel that was stained with Coomassie blue. The molecular masses of the protein marker (Invitrogen) are given, in kilodaltons.

In order to assess the impact of mutations on nsp14’s enzymatic activities, we set out to purify recombinant MERS-CoV nsp14 and develop an *in vitro* ExoN assay. Thus far, such an assay had been described only for the equivalent SARS-CoV protein (19, 20, 26, 71). Wild-type and mutant MERS-CoV nsp14 proteins carrying an N-terminal His tag were expressed in Escherichia coli Rosetta(DE3) pLysS. Proteins were purified by immobilized metal affinity chromatography (IMAC) followed by size exclusion chromatography. Upon SDS-PAGE, the purified MERS-CoV nsp14 was consistently detected as a doublet (with the lower band being most abundant), migrating at the expected molecular mass of ∼55 kDa (Fig. 6). As a positive control, we purified SARS-CoV nsp14 (26) and used it during optimization of the enzymatic assays for MERS-CoV nsp14. The substrate used for ExoN activity assays was a 5′ ^32^P-labeled 22-nucleotide (nt) long synthetic RNA, as previously used in similar assays with SARS-CoV nsp14 (referred to as oligonucleotide H4 in reference 26). Nucleotides 5 to 22 of this substrate are predicted to form a hairpin with a stem consisting of seven G-C base pairs and a loop of 4 As (26), while the remaining 4 nucleotides form a 5′-terminal single-stranded tail.

**FIG 7:**
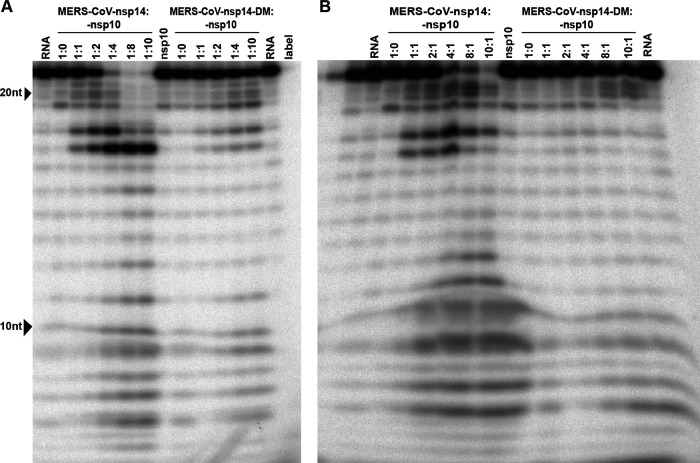
Optimization of MERS-CoV nsp14 *in vitro* ExoN assay conditions. The substrate for the assay was a 22-nt synthetic RNA (H4) that was ^32^P labeled at its 5′ terminus. Cleavage products were separated by polyacrylamide gel electrophoresis and visualized by autoradiography. (A) Analysis of ExoN activity in the presence of increasing amounts of nsp10, using wt MERS-CoV-nsp14 (left) and the ExoN double-knockout mutant (DM; D90A/E92A; right). The RNA substrate was hydrolyzed for 90 min at 37°C using a fixed concentration of nsp14 (200 nM) and increasing nsp10 concentrations, ranging from 0 to 1,600 nM. (B) Evaluation of the ExoN activity of increasing concentrations (200 to 2,000 nM) of wt or DM nsp14 in the presence of a fixed amount of nsp10 (200 nM).

**FIG 8:**
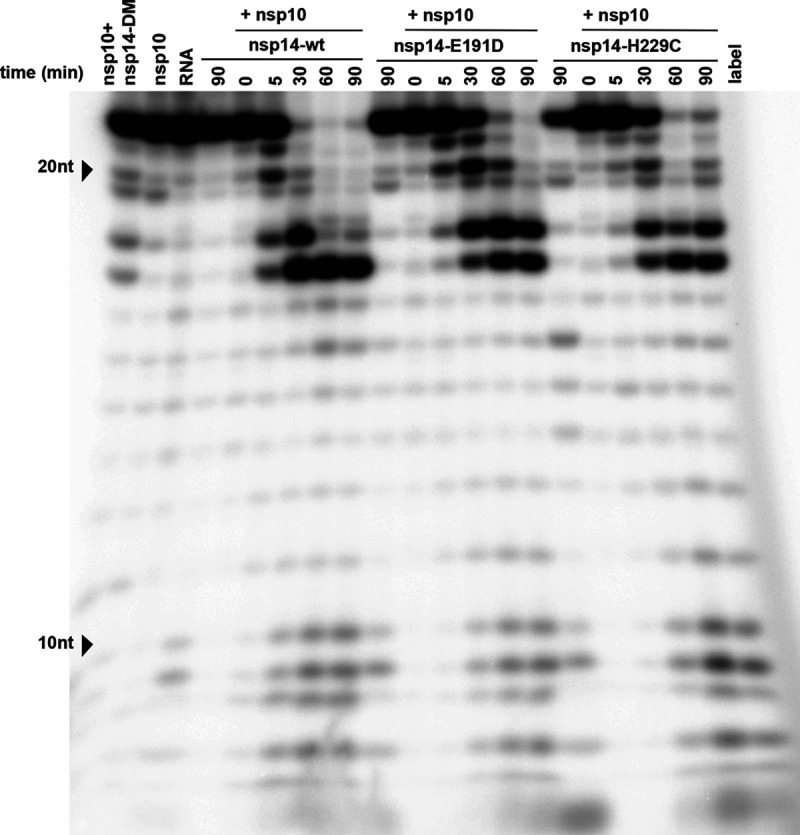
Time course analysis of the *in vitro* ExoN activity of MERS-CoV nsp14. The ExoN activity of different recombinant nsp14 proteins (wt, D90A/E92A, E191D, and H229C) was evaluated by incubating 200 nM nsp14 and 800 nM nsp10 for 0, 5, 30, 60, and 90 min at 37°C. As controls, individual proteins (800 nM) were incubated for 90 min. For technical details, see the legend to Fig. 7.

Previously, the ExoN activity of SARS-CoV nsp14 was found to be dramatically stimulated by the addition of nsp10 as a cofactor (26). Consequently, we also expressed and purified MERS-CoV nsp10 and optimized the ExoN assay by testing different molar ratios between nsp14 and nsp10 (Fig. 7A, left) and different nsp14 concentrations (Fig. 7B, left) and by using different incubation times (Fig. 8, left). MERS-CoV nsp14 ExoN activity was found to be stimulated by nsp10 in a dose-dependent manner (Fig. 7A), while nsp10 did not exhibit any nuclease activity by itself (Fig. 7B, nsp10 lane). The full-length substrate was more completely degraded when a 4-fold (or higher) excess of nsp10 over nsp14 was used, compared to the effect of merely increasing the nsp14 concentration in the assay (Fig. 7B). Different substrate degradation patterns were observed when nsp14 alone was compared with the nsp14-nsp10 in complex, which likely derived from structural and functional differences between these two nsp14 conformations. Similar observations were previously reported for SARS-CoV nsp14 (20, 21, 26) and recently for SARS-CoV-2 nsp14 (72). When an excess of nsp14 over nsp10 was used, an intermediate (or mixed) pattern of degradation products was obtained (Fig. 7B), including an elevated amount of products with lengths of 21 to 17 and 11 to 6 nt. Introduction of the D90A/E92A motif I double substitution resulted in a major reduction of ExoN activity, although a certain level of residual activity was observed, in particular when large amounts of nsp14 (Fig. 7B, right) or a relatively high nsp10-to-nsp14 ratio (Fig. 7A, right) was used. Similar observations were previously made for SARS-CoV nsp14 (20, 26).

**FIG 9:**
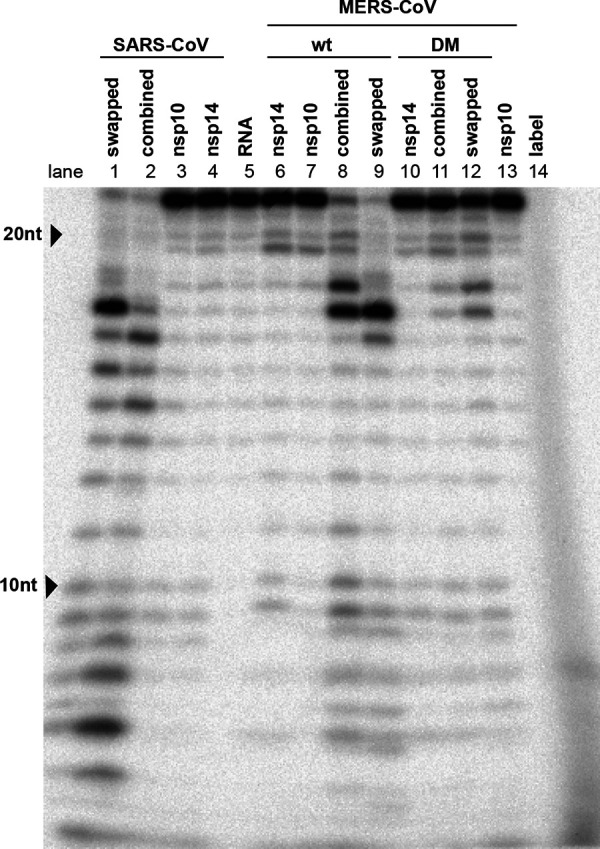
Cross-activation of the *in vitro* activity of SARS-CoV and MERS-CoV nsp14 by heterologous nsp10. The nsp10 cofactor was exchanged in ExoN assays performed with MERS-CoV and SARS-CoV nsp14, using a 1:4 ratio between nsp14 and nsp10 and a 90-min incubation at 37°C. For technical details, see the legend to Fig. 7.

Using a 4:1 ratio of nsp10 to nsp14, MERS-CoV ExoN activity was analyzed in a time course experiment (Fig. 8). Over time, the full-length substrate was progressively converted to a set of degradation products in the size range of 6 to 18 nt. We anticipated that the structure of the H4 RNA substrate would change from a duplexed to a single-stranded conformation, upon digestion of one side of the hairpin’s stem by ExoN’s nuclease activity. As the ExoN enzymes of other CoVs were reported to prefer dsRNA substrates (19, 71), the degradation of the substrate might be slowed down substantially after removal of the first two nucleotides from its 3′ end (26). This would explain the abundant generation of degradation products of 16 and 17 nt (Fig. 7 and 9) and suggest that the preference for dsRNA substrates is indeed shared by MERS-CoV ExoN.

Degradation of the RNA substrate could be observed within 5 min, and the full-length substrate was essentially gone after 30 min. A similar reaction with the nsp14 double mutant resulted in only a small amount of substrate degradation after 90 min (Fig. 8, leftmost lane). Taken together, our results convincingly demonstrate the *in vitro* 3′-to-5′ exonuclease activity of purified MERS-CoV nsp14. As in the case of the SARS-CoV enzyme, nsp10 is a critical cofactor that can strikingly upregulate MERS-CoV ExoN activity *in vitro*.

### MERS-CoV nsp10 modulates nsp14 ExoN activity.

In order to investigate differences that might explain the variable phenotype of CoV ExoN knockout virus mutants, we compared ExoN activities between SARS-CoV and MERS-CoV nsp14, using the optimized *in vitro* assay described above. An incubation time of 90 min was used, unless indicated otherwise. The nsp14 and nsp10 preparations of both viruses were first tested individually in an assay containing the H4 RNA substrate and Mg^2+^ ions (26, 73, 74). As expected, this revealed only traces of exonuclease activity for both nsp14 proteins alone (Fig. 9, lanes 4 and 6). When the two proteins were combined in the same reaction, a strong increase of ExoN activity was observed for both nsp14-nsp10 pairs, with the SARS-CoV pair appearing to be somewhat more active than the MERS-CoV pair (Fig. 9, lanes 2 and 8).

The exchange of the SARS-CoV and MERS-CoV nsp10 cofactors revealed that they can cross-activate the ExoN activity of nsp14 from the other virus, although some changes in the pattern of degradation products were observed (Fig. 9, lanes 1 and 2 and lanes 8 and 9). However, the residual ExoN activity of the motif I double mutant (DM) apparently was not affected by the choice of nsp10 cofactor (Fig. 9, compare lanes 11 and 12). The observed subtle changes in degradation product patterns are another indication that nsp10 modulates nsp14 ExoN activity, presumably using interaction surfaces that are well conserved across CoV genera (73, 74).

### ExoN activity of MERS-CoV active-site and H229C mutants.

**FIG 10:**
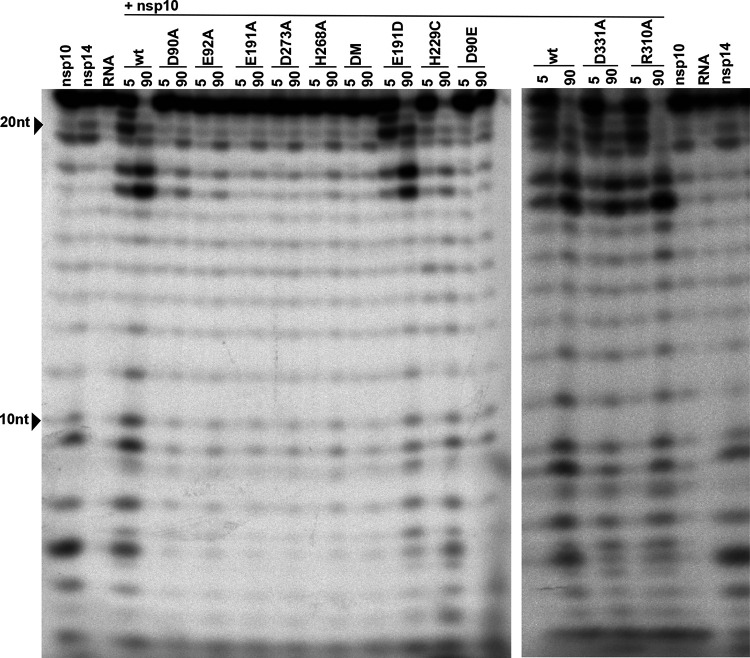
*In vitro* ExoN activity of MERS-CoV nsp14 mutants. Residues from the DEDDh catalytic motif and ZF1 motif of the nsp14 ExoN domain and the nsp14 N7-MTase domain were mutated as indicated. Assays were performed using a 1:4 ratio between nsp14 and nsp10 and a 90-min incubation at 37°C. For technical details, see the legend to Fig. 7.

Having established the optimal conditions for MERS-CoV ExoN *in vitro* activity, we evaluated the impact of a subset of the DEEDh active-site mutations that were used during our reverse genetic analysis (Fig. 2A). For each mutant tested, two protein batches were purified and analyzed independently in duplicate using the same batch of MERS-CoV nsp10 for all assays. As can be seen in Fig. 10, replacement with Ala of each of the five active-site residues resulted in a nearly complete loss of ExoN activity, with the D90A, E92A, and H268A substitutions appearing to be slightly less detrimental than E191A and D273A. A clearly different result was again obtained with the E191D mutant, which displayed an activity level comparable to that of wt nsp14, corresponding to the properties of the corresponding virus mutant (Fig. 2, 4, and 5). Overall, the severe impact of active-site mutations on ExoN activity was fully in line with the nonviable phenotypes observed for the same mutants when they were tested using reverse genetics (Fig. 2).

We also evaluated the impact of the H229C ZF1 mutation, which—despite its conservative nature—yielded a crippled mutant virus (Fig. 2), and of two N7-MTase mutations (see below). The N7-MTase mutants displayed wt nsp14-like ExoN activities (Fig. 10), suggesting that, as in SARS-CoV nsp14, ExoN and N7-MTase activities are functionally separated (36). Analyzing the substrate degradation pattern of the H229C mutant (Fig. 8) revealed that the enzyme was less efficient in its ability to degrade RNA than wt nsp14. This can be seen, for example, when the reaction products after 5-min assays are compared (Fig. 8 and 10). A more detailed quantitative assessment of the activity level of nsp14 mutants is beyond the scope of this study. The H229C mutation clearly reduced ExoN activity *in vitro*, potentially by affecting the structure of the ExoN domain, as ZF1 is in close proximity to the nsp10 interaction surface (20). However, a similar reduction of ExoN activity was observed for the E191D mutant (Fig. 8), which was much more viable than the H229C mutant in the context of our reverse genetics studies. This suggests that the H229C replacement may affect additional functions or interactions of the ExoN domain that are important for viral RNA synthesis and viability.

### ExoN mutations do not interfere with N7-MTase activity *in vitro*.

The nsp14 N7-MTtase activity is deemed essential for formation of a functional RNA cap, enabling the translation of CoV mRNAs and protecting them from degradation. Consequently, at least in theory, ExoN mutations could also be detrimental to virus replication if they somehow affect the crucial enzymatic activity of the other nsp14 domain. In order to evaluate this possibility, the same recombinant protein preparations used for the ExoN assays (Fig. 6) were evaluated in an N7-MTase biochemical assay using the synthetic cap analogues GpppA and m^7^GpppA (control) as substrates. Moreover, nsp14 R310A and D331A mutants were used as negative controls in view of their predicted involvement in the binding of the triphosphate moiety of the RNA chain and the methyl donor (S-adenosylmethionine [SAM]), respectively (20, 26, 36). In this assay, nsp14 can methylate GpppA by transferring the [^3^H]CH_3_ moiety provided by [^3^H]SAM. The resulting radiolabeled m^7^GpppA product can be quantified using a DEAE filter-binding assay, followed by liquid scintillation counting and data normalization against the activity of wt control protein (25).

**FIG 11:**
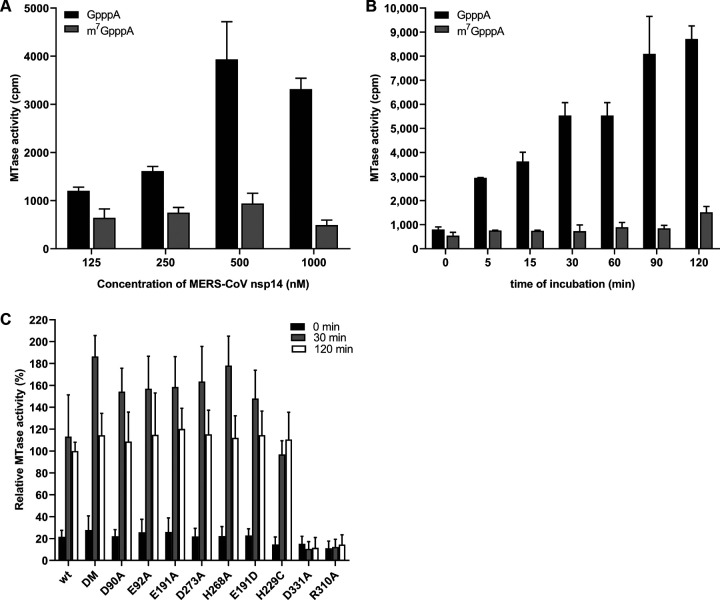
*In vitro* N7-MTase activity of MERS-CoV nsp14 mutants. The N7-MTase activity of recombinant nsp14 was analyzed *in vitro* by filter binding assay using synthetic cap analogues as the substrate. (A) Increasing concentrations of MERS-CoV nsp14 were incubated with GpppA and m^7^GpppA in the presence of [^3^H]SAM for 30 min. (B) The ability of nsp14 to methylate GpppA or m^7^GpppA was determined after reaction times between 0 and 120 min at 30°C. (C) The ability of nsp14 mutants to methylate GpppA was measured four times in duplicate. Values were normalized to the wt control (*n* = 8; means ± SD).

Recombinant MERS-CoV nsp14 was found to methylate GpppA, but not m^7^GpppA (Fig. 11A), which yielded a signal that was similar to the background signal in assays lacking nsp14 or substrate (data not shown). Methylation increased with time and reached a plateau after 120 min (Fig. 11B). The N7-MTase activity of the various nsp14 mutants was compared with that of wt nsp14 after reaction times of 30 and 120 min (Fig. 11C). While the R310A and D331A control mutations fully inactivated the N7-MTase activity of MERS-CoV nsp14, none of the ExoN active-site mutations tested was found to alter the enzyme’s activity. These results again support the notion that ExoN and N7-MTase domains are functionally separated, as previously demonstrated for SARS-CoV nsp14 (36). We therefore conclude that the lethal impact of ExoN inactivation on MERS-CoV replication (Fig. 2A) cannot be attributed to inadvertent effects on the activity of the N7-MTase domain that is present in the same nsp14 replicase subunit.

## DISCUSSION

In this study, we demonstrate that the impact of ExoN inactivation on virus viability and RNA synthesis distinguishes MERS-CoV and SARS-CoV-2 from two other betacoronaviruses, MHV and SARS-CoV. Whereas ExoN inactivation in the latter two viruses yields viable mutants that are only mildly crippled and exhibit a 'mutator phenotype' (22, 23, 29), both conservative and alanine substitutions of MERS-CoV ExoN catalytic residues abolished the recovery of infectious progeny (Fig. 2) and the detection of viral RNA synthesis (Fig. 4). The only exception was the conservative E191D mutant, which was found to exhibit near-wt ExoN activity (Fig. 8 and 10). Though the study was limited in scope, it is even more remarkable that our mutagenesis of SARS-CoV-2 yielded a very similar nonviable phenotype for an ExoN knockout mutant, in spite of the close relationship of this virus with SARS-CoV, ExoN knockout mutants of which are crippled but quite viable (Fig. 3). For most MERS-CoV ExoN knockout mutants and for the SARS-CoV-2 double mutant, immunofluorescence microscopy revealed some virus-specific signal in a few individual cells at 2 dpt (Fig. 2C and 3B), a time point at which wt viruses have already spread efficiently and infected all susceptible cells in the culture. The very limited labeling observed for mutant virus-transfected cultures is difficult to interpret and requires further analysis, but it suggests that viral RNA synthesis is not completely abrogated, at least not in all transfected cells, and likely explains how reversion occurred occasionally for several of the MERS-CoV single mutants late in the experiment.

Based on nsp14 conservation (Fig. 1) and the viable phenotype of SARS-CoV and MHV ExoN-knockout mutants, MERS-CoV and SARS-CoV-2 were expected to tolerate ExoN inactivation, in particular since the enzyme was proposed to improve the fidelity of CoV replication without being essential for RNA synthesis *per se* (10, 14, 21–23, 26, 29). This notion is further supported by the fact that the CoV RdRp (nsp12) exhibits *in vitro* activity in the absence of nsp14 (27). We therefore anticipated that an excess of deleterious mutations would first have to accumulate before becoming detrimental. Contrary to these expectations, an immediate lack of viability was observed when MERS-CoV or SARS-CoV-2 ExoN knockout mutants were launched. It is noteworthy that similar observations were previously made for the corresponding ExoN knockout mutants of the alpha-CoVs HCoV-229E (19) and TGEV (34) and the gamma-CoV avian infectious bronchitis virus (IBV) (E. Bickerton, S. Keep, and P. Britton, personal communication). Furthermore, in line with our observations, a recent report from the Denison laboratory (awaiting peer review) briefly mentions that in their hands, nsp14-ExoN catalytic mutants also could not be rescued for both MERS-CoV and SARS-CoV-2 (30).

None of the ExoN mutations tested had a negative effect on the *in vitro* activity of the N7-MTase domain of nsp14, which is deemed essential for viral mRNA capping (Fig. 11). This is consistent with previous observations for SARS-CoV nsp14, in which the ExoN and N7-MTase activities were shown to be functionally distinct but structurally interconnected by a hinge region that confers flexibility (21, 36). Given their unchanged N7-MTase activity, the nonviable phenotype of MERS-CoV ExoN active-site mutants must be attributed to a negative effect on an additional and apparently critical function of the ExoN domain, which likely is directly involved in primary RNA synthesis rather than in (longer-term) fidelity control. At present we cannot explain why SARS-CoV and MHV ExoN knockouts can apparently tolerate ExoN active-site substitutions that are lethal to five other CoVs (including the closely related SARS-CoV-2). Within the betacoronavirus group, in our experience, SARS-CoV and MHV display the most robust RNA synthesis and replication in cell culture compared to MERS-CoV and SARS-CoV-2 (75–77), as also illustrated by the SARS-CoV and SARS-CoV-2 plaque phenotypes presented in Fig. 3. Possibly, the recovery of viable progeny depends on reaching a minimum level of RNA synthesis, which may somehow be achieved by only the most efficiently replicating CoVs. Admittedly, even bearing this possibility in mind, it remains difficult to reconcile the 1- to 2-log reduction of progeny titers observed for MHV and SARS-CoV ExoN knockout mutants with the complete loss (>6-log reduction) of infectious progeny observed for the ExoN knockout mutants of the other CoVs.

In order to eliminate technical issues that might somehow prohibit the successful recovery of MERS-CoV ExoN knockout mutants and explain the phenotypic differences with other CoVs, we explored various details of the transfection protocol. This included the use of a DNA-launched system, similar to that used for TGEV (34), and the propagation of progeny virus in both innately immunocompetent and -incompetent cells (Huh7 and Vero cells, respectively). However, this did not change the negative outcome of our transfection experiments, which were repeated more than 10 times for several of the nonviable mutants, always using wt and E191D MERS-CoV as positive controls that proved to be consistently viable. Next, we performed experiments in which BHK-21 cells were cotransfected with a synthetic mRNA expressing the N protein, which has been reported to promote the recovery of recombinant CoVs (60, 61). Indeed, judging from immunolabelings and early virus yields, such an effect could be observed, but it did not alter the outcome for the nonviable ExoN knockout mutants, and 3-dpt virus harvests continued to yield no plaques for both MERS-CoV and SARS-CoV-2. These combined observations strengthen our conclusion that—in addition to its proposed role as a proofreading enzyme—ExoN likely has an additional role in CoV RNA synthesis (63).

As reported for MHV and SARS-CoV ExoN mutants (23, 78), possible (late) reversion was observed for a few of our MERS-CoV ExoN active-site mutants, specifically, those with E191A, D273E, D273A, and in particular D90E, which had reverted by 6 dpt in four of eight experiments. Together with the immunolabeling results presented in Fig. 2C, this suggests that these mutants (and perhaps others as well) exhibit a low residual level of RNA synthesis that is the basis for these low-frequency reversion events. Furthermore, in follow-up studies with the crippled H229C ZF1 mutant, a possible pseudorevertant carrying a second-site mutation (Q19R) in nsp8 was identified in three independently obtained progeny samples, providing genetic support for an interaction between nsp8 and nsp14, which may be relevant in the context of the association of nsp14 with the tripartite RdRp complex consisting of nsp7, nsp8, and nsp12 (21, 27, 79–81). Future studies will address the properties of these nsp14 ExoN knockout mutants and their (pseudo)revertants in more detail.

In the only viable MERS-CoV ExoN active-site mutant obtained, E191D, the catalytic motif was changed from DEEDh to DEDDh, which is characteristic of all members of the exonuclease family that ExoN belongs to (14, 15, 82). The growth of the E191D virus mutant was comparable to that of wt virus (Fig. 5). Biochemical assays revealed that the E191D-ExoN enzyme is able to hydrolyze a dsRNA substrate with an activity level approaching that of the wt protein (Fig. 8 and 10). Although the E191D mutant was somewhat more sensitive to the mutagenic agent 5-FU (Fig. 5C and D) (28, 78), its ExoN activity does not appear to be dramatically altered by this conservative substitution in the active site.

For this study, we developed an *in vitro* assay to evaluate MERS-CoV ExoN activity using a largely double-stranded RNA substrate (Fig. 7, 8, 9, and 10). As previously observed for SARS-CoV nsp14 (26), MERS-CoV ExoN activity was strongly enhanced by the presence of nsp10 (Fig. 7), in line with the formation of an nsp10:nsp14 heterodimer, as observed in biochemical and structural studies (20, 26, 83). Slightly different patterns of degradation of the H4 substrate were observed when the SARS-CoV and MERS-CoV ExoN enzymes were compared *in vitro*. Likewise, the exchange of the nsp10 cofactor for the nsp10 subunit of the other virus, using the same substrate and the same nsp10:nsp14 ratio (1:4) (Fig. 9), resulted in a somewhat different pattern of substrate degradation, suggesting minor differences in the interaction of the nsp10:nsp14 complex with this particular RNA substrate. Previously, it was demonstrated that nsp10 is interchangeable between CoV subgenera in its role as cofactor for the nsp16 2′-O-methyltransferase, which was attributed to the high level of conservation of the nsp10-nsp16 interaction surface (84). As nsp14 and nsp16 share an interaction surface on nsp10 (21, 26, 73), we explored whether a similar cofactor exchange was possible in the context of nsp14’s ExoN activity, which was indeed found to be the case (Fig. 9). Structurally, nsp14 interaction with nsp10 is figuratively similar to a “hand (nsp14) over fist (nsp10)” conformation (21). Upon formation of this complex, nsp10 induces conformational changes in the N-terminal region of ExoN that adjust the distance between the catalytic residues in the back of the nsp14 palm and, consequently, impact ExoN activity (21). The exchange of the nsp10 cofactor between the two beta-CoVs might affect this conformation and, consequently, modulate the ExoN activity of the nsp14:nsp10 complex.

Alanine substitutions of active-site residues severely reduced but did not completely abrogate the *in vitro* activity of MERS-CoV ExoN (Fig. 7 to 10), as previously shown for certain SARS-CoV nsp14 mutants (20, 36) and recently also for SARS-CoV-2 (72). Based on the two-ion-metal catalytic mechanism underlying the exonuclease activity of DEDDh family members (17, 62) and the SARS-CoV nsp14 structure, it was predicted that the various ExoN motifs contribute differently to the excision of nucleoside monophosphates (20, 21). Mutation of ExoN catalytic residues can alter ion binding (31) or disturb the fragile chemical equilibrium, as shown for conservative mutations (corresponding to E191D and D273E) in the Klenow fragment, a member of the DEDDh exonuclease family, which reduced ExoN activity by >96% (85). In general, all DEEDh mutations that yielded nonviable virus mutants exhibited similarly low levels of residual ExoN activity *in vitro* (Fig. 10), indicating that each of these residues is important for catalysis.

Our study suggests that, in addition to the active-site residues, also other motifs in MERS-CoV ExoN are important for virus viability, specifically the two ZF motifs that were probed using two point mutations each (Fig. 2A). In previous ZF1 studies, a mutation equivalent to H229A caused solubility issues during expression of recombinant SARS-CoV nsp14 (20) and resulted in a partially active ExoN in the case of white bream virus, a tobanivirus that also belongs to the order *Nidovirales* (86). It was suggested that ZF1 contributes to the structural stability of ExoN, as it is close to the surface that interacts with nsp10 (20). Here, we demonstrate that the more conservative H229C replacement, which converts ZF1 from a nonclassical CCCH-type ZF motif into a classical CCCC type (69), was tolerated during recombinant protein expression and yielded an ExoN that is active *in vitro* (Fig. 8). This likely contributed to the fact that the H229C virus mutant retained a low level of viability (Fig. 2 and data not shown). Nevertheless, its overall crippled phenotype and the nonviable phenotype of the C201H mutant clearly highlighted the general importance of ZF1 for virus replication. In contrast, the corresponding TGEV mutant (ZF-C) was not strongly affected and could be stably maintained over several passages (34) The reverse genetics data suggest that ZF2, which is in close proximity to ExoN catalytic residues (20), is equally important, although technical complications with expression of the C261A and H264R nsp14 mutants prevented us from performing *in vitro* activity assays.

Like the ExoN domain of the arenavirus nucleoprotein (32, 33), the CoV ExoN was proposed to be involved in innate immune evasion (34, 35, 87), possibly by degrading viral dsRNA that in the case of CoVs is confined to characteristic double-membrane vesicles (88–90). For TGEV, this suggestion was based on the reduced accumulation of dsRNA by the ZF-C mutant, which, however, remains to be characterized in more detail. In the absence of a TGEV ExoN activity assay, and in view of our data for the equivalent MERS-CoV ZF1 mutant, it seems premature to assume that the reduced levels of dsRNA in infected cells are caused by increased exonuclease activity of the ZF-C ExoN mutant (34).

In general, the properties of viable CoV ExoN mutants warrant further analysis. In future studies, the repertoire of residues probed by site-directed mutagenesis could be extended beyond active site and ZF motifs, which may help in particular to establish how directly reduced ExoN activity, primary viral RNA synthesis, and enhanced innate responses are interconnected. Regardless of its possible interactions with host cell pathways, nsp14 clearly is a key subunit of the multienzyme complex that drives CoV genome replication, subgenomic RNA synthesis, and RNA recombination. Understanding the structure-function interplay between ExoN and other (viral and/or host) components will be key to elucidating its role in CoV RNA synthesis and evolution (91, 92). Taking into account the current SARS-CoV-2 pandemic, understanding the phenotypic differences between ExoN knockout mutants of different CoVs may contribute to the design of improved antiviral approaches, including those relying on “lethal mutagenesis” or direct interference with viral RNA synthesis.

## MATERIALS AND METHODS

### Cell culture.

Baby hamster kidney cells (BHK-21; ATCC CCL10), Vero E6 cells, Vero (ATCC CCL81) cells, and HuH7 cells were cultured as described previously (76, 93). Vero and Vero E6 cells were kindly provided by the Department of Viroscience, Erasmus Medical Center, Rotterdam, the Netherlands, and HuH7 cells by Ralf Bartenschlager, Heidelberg University, Germany. For transfections, cells were maintained in Eagle’s minimal essential medium (EMEM; Lonza) with 8% fetal calf serum (FCS; Bodinco) supplemented with 100 IU/ml of penicillin and 100 μg/ml of streptomycin (Sigma) and 2 mM l-glutamine (PAA Laboratories) and incubated at 37°C with 5% CO_2_. Infection of Vero, Vero E6, and HuH7 cells was carried out in EMEM containing 2 % FCS. SARS-CoV and SARS-CoV-2 were grown in Vero E6 cells and MERS-CoV in Vero and HuH7 cells.

### Reverse genetics.

Mutations in the MERS-CoV nsp14-coding region were engineered in a bacterial artificial chromosome (BAC) vector (52, 53) containing a full-length cDNA copy of MERS-CoV strain EMC/2012 (44, 54), by two-step *en passant* recombineering in E. coli (94). For SARS-CoV and SARS-CoV-2, a BAC DNA vector containing a full-length cDNA copy of the SARS-CoV Frankfurt-1 sequence (95) or SARS-CoV-2 BetaCoV/Wuhan/IVDC-HB-01/2019 (59, 96) was used. When the primers were designed, a translationally silent marker mutation was introduced near the site of mutagenesis in order to differentiate between the occurrence of reversion and (possible) contamination with parental virus. For each mutation, two mutant BACs were isolated independently, the nsp14-coding region was verified by sequencing, and both BACs were used for *in vitro* runoff transcription and virus launching.

Approximately 5 μg of BAC DNA was linearized with NotI, and full-length RNA was obtained by *in vitro* transcription with T7 RNA polymerase followed by lithium chloride precipitation according to the manufacturer's protocol (mMessage-mMachine T7 kit; Ambion). For some of the MERS-CoV and the SARS-CoV-2 transfections, synthetic mRNAs expressing the respective N proteins were generated as described previously (60, 61) and 10 μg was cotransfected with the corresponding full-length RNA. To this end, 5 μg of RNA was electroporated into 5 × 10^6^ BHK-21 cells (for MERS-CoV) or BHK-21 cells expressing SARS-CoV N protein (for SARS-CoV and SARS-CoV-2) (97) using the Amaxa Nucleofector 2b (program A-031) and Nucleofection T solution kit (Lonza). Transfected cells were mixed with HuH7 or Vero cells (for MERS-CoV) or Vero E6 cells (for SARS-CoV and SARS-CoV-2) in a 1:1 ratio and plated for supernatant harvesting, intracellular RNA isolation, and analysis by immunofluorescence microscopy. Immunolabeling was performed as described before (76), using antibodies recognizing double-stranded RNA (dsRNA) (98) or SARS-CoV nsp4 (77, 99).

Cells were incubated at 37°C up to a maximum of 6 days posttransfection (dpt). Supernatants were collected when full cytopathic effect was observed, at 3 dpt or at the end of the experiment. Virus titers were determined by plaque assay in HuH7 and Vero cells (100). In order to confirm the presence of engineered mutations in viral progeny, HuH7 and Vero cells were infected with supernatants harvested from transfected cells and intracellular RNA was isolated at 18 h postinfection as described above. cDNA was synthesized by reverse transcription using RevertAid H minus reverse transcriptase (Thermo Fisher Scientific) and random hexamer primers (Promega), in combination with a primer targeting the 3′ end of the viral genome. The full-length genome or the nsp14-coding region was amplified by PCR using MyTaq DNA polymerase (Bioline), and after purification, the PCR product was sequenced by Sanger sequencing. Genome sequencing by NGS was performed as described before (77). All work with live (recombinant) class-3 CoVs was done in a biosafety level 3 laboratory at Leiden University Medical Center.

### Analysis of viral RNA synthesis.

Isolation of intracellular RNA was performed by lysing infected cell monolayers with TriPure isolation reagent (Roche Applied Science) according to the manufacturer’s instructions. After purification, intracellular RNA samples were loaded onto a 1.5% agarose gel containing 2.2 M formaldehyde, which was run overnight at low voltage in MOPS buffer (10 mM morpholinepropanesulfonic acid [sodium salt] [pH 7], 5 mM sodium acetate, 1 mM EDTA). Dried agarose gels were used for direct detection of viral mRNAs by hybridization with a ^32^P-labeled oligonucleotide probe (5′-GCAAATCATCTAATTAGCCTAATC-3′) that is complementary to the 3′-terminal sequence of MERS-CoV genome and all subgenomic mRNAs. After hybridization, RNA bands were visualized (using exposure times of up to 28 days) and quantified by phosphorimaging using a Typhoon-9410 variable-mode scanner (GE Healthcare) and ImageQuant TL software (GE Healthcare).

PCR primers and TaqMan probes targeting ORF1a (junction of the nsp2-nsp3 coding region), the nucleocapsid (N) protein gene, or the leader-body transcription-regulatory sequence (TRS) junction of subgenomic mRNA3 were designed and analyzed for multiplex quality using Beacon Designer software (Premier Biosoft). Reverse transcription (RT) was performed using RevertAid H minus reverse transcriptase (Thermo Fisher Scientific) and a mix of specific reverse primers targeting ORF1a, ORF8, or subgenomic RNA 3 (primer sequences are available upon request). The mRNA derived from the cellular β-actin gene was used as a reference housekeeping gene. Tagged primers were used to differentiate between positive- and negative-stranded viral RNA. Samples were assayed by TaqMan multiplex real-time PCR using TaqMan Universal Master Mix II and a CFX384 Touch real-time PCR detection system (Bio-Rad). A standard curve was obtained using an *in vitro* transcript derived from a synthetic plasmid that contained all PCR targets. cDNA was obtained as described above. Each RNA sample was analyzed in triplicate.

### Plaque reduction assay.

HuH7 cells seeded in 6-well clusters were infected with recombinant MERS-CoV at low MOI (30 PFU/well) for 1 h at 37°C. Subsequently, the inoculum was replaced with 2 ml of a 1.2% suspension of Avicel (RC-581; FMC Biopolymer) (101) in Dulbecco’s minimal essential medium (DMEM) (containing 2% FCS and antibiotics) and serial dilutions of 5-FU (F6627; Sigma-Aldrich) or ribavirin (R9644; Sigma-Aldrich) ranging from 0 to 400 μM. Cells were incubated at 37°C for 72 h and fixed with 7.4% formaldehyde, and plaques were visualized using crystal violet staining.

To compare the effect of 5-FU treatment on the progeny titers of wt and nsp14-E191D rMERS-CoV, confluent monolayers of HuH7 were incubated for 30 min at 37°C with solvent or a range of 5-FU concentrations. The drug was then removed and cells were infected at an MOI of 0.1 for 1 h at 37°C. After removal of the inoculum, EMEM containing 2% FCS and solvent or a matching concentration of 5-FU was added to the wells. Supernatants were collected after 30 h, and rMERS-CoV progeny titers were determined by plaque assay. All drug-treated samples were normalized to the untreated vehicle control, and values were expressed as fold change compared to untreated-virus titers.

### Expression and purification of recombinant CoV nsps.

SARS-CoV nsp10 and nsp14 were produced as described before (25) and used as positive controls in all biochemical assays. MERS-CoV nsp10 was expressed using pET30a vector and purified as described previously (84, 102). All MERS-CoV nsp14 constructs were cloned into expression vector pDEST14 with an N-terminal His_6_ tag using the Gateway system (25). MERS-CoV nsp14 mutant expression plasmids were generated by QuikChange site-directed mutagenesis using Accuzyme DNA polymerase (Bioline) following the manufacturer’s instructions. pDEST14 plasmids expressing MERS-CoV nsp14 were transformed into competent E. coli strain Rosetta(DE3)pLysS (Novagen) and cultured in Luria-Bertani (LB) broth supplemented with 100 μg/ml of ampicillin and 30 μg/ml of chloramphenicol. Protein expression was induced at an optical density at 600 nm (OD_600_) of 0.8 by adding 50 μM isopropyl-β-d-α-thiogalactopyranoside (IPTG; Bioline). After 24 h at 13°C, induced cells were harvested and lysed in a buffer containing 50 mM Tris-HCl (pH 7.5), 150 mM NaCl, 5 mM β-mercaptoethanol, 5% glycerol, 1 mM phenylmethylsulfonyl fluoride (PMSF), and 20 mM imidazole (103). Next, the lysate was centrifuged at 12,000 × *g* for 30 min, and the soluble fraction was column purified by immobilized metal ion affinity chromatography using nickel Sepharose high-performance beads (17526802; GE Healthcare). The eluate was fractionated by gel filtration on a Superdex-200 Increase 10/300GL column (GE Healthcare) in buffer containing 30 mM HEPES (pH 7.5), 300 mM NaCl, and 5% glycerol. Finally, proteins were concentrated using ultrafiltration devices with a molecular mass cutoff of 30 kDa (Millipore), and protein concentrations were measured using spectrophotometry. All purified proteins were analyzed by SDS-PAGE followed by Coomassie blue staining as well as by Western blotting using a mouse monoclonal antibody against the His_6_ tag (Novagen). Protein aliquots were stored at −80°C in 50% glycerol (vol/vol) and used for enzymatic assays.

### Exonuclease activity assay.

Synthetic RNA H4 (26) was radiolabeled at its 5′ end using T4 polynucleotide kinase (Epicentre) and [γ-^32^P]ATP (Perkin Elmer) (77). Unless stated otherwise in figures or legends, reaction mixtures contained 200 nM recombinant nsp14, 800 nM nsp10, and 750 nM radiolabeled substrate in 40 mM Tris-HCl (pH 7.5) containing 5 mM MgCl_2_ and 1 mM dithiothreitol (DTT). After incubation at 37°C for up to 90 min, reactions were stopped by addition of an equal volume of loading buffer containing 96% formamide and 10 mM EDTA. Samples were then loaded on 7 M urea-containing 20% (wt/vol) polyacrylamide gels (acrylamide/bisacrylamide ratio, 19:1) buffered with 0.5× Tris-borate-EDTA and run at high voltage (1,600 V). Results were visualized by phosphorimaging as described above.

### N7-methyltransferase activity assay.

Methyltransferase assays were performed in 40 mM Tris-HCl (pH 8.0), 5 mM DTT, 2 μM ^7Me^GpppA or GpppA RNA cap analogue (New England Biolabs), 10 μM adenosyl-methionine (AdoMet; Thermo Fisher), 0.03 μCi/μl [^3^H]AdoMet (PerkinElmer) (25). For each reaction, MERS-CoV or SARS-CoV nsp14 was added to a final concentration of 500 or 250 nM, respectively. Reaction mixtures were incubated at 30°C for up to 120 min, and reactions were stopped by the addition of a 10-fold volume of 100 μM ice-cold adenosyl-homocysteine (AdoHcy; Thermo Fisher). Then, samples were spotted on a DEAE filter mat (PerkinElmer) prewet with Tris-HCl (pH 8.0) buffer. Filter mats were washed twice with 10 mM ammonium formate (Sigma-Aldrich) (pH 8.0), twice with MilliQ water, and once with absolute ethanol (Sigma-Aldrich). After air drying for 10 min, filter mats were cut, and relevant pieces were transferred to individual tubes. Betaplate scintillation fluid (PerkinElmer) was added, and the amount of ^3^H label bound was measured in counts per minute using a Wallac scintillation counter. For relative quantification, incorporation measurements for mutant proteins were normalized to values obtained with the wt control nsp14. Samples were measured in duplicate in each experiment.
